# ATF2 loss promotes 5-FU resistance in colon cancer cells via activation of the ATR-Chk1 damage response pathway

**DOI:** 10.1186/s12885-023-10940-0

**Published:** 2023-05-27

**Authors:** Hao Yang, Kerstin Huebner, Chuanpit Hampel, Katharina Erlenbach-Wuensch, Selva Babu Selvamani, Vikas Shukla, Carol I. Geppert, Arndt Hartmann, Vijayalakshmi Mahadevan, Regine Schneider-Stock

**Affiliations:** 1 Experimental Tumorpathology, University Hospital Erlangen, Friedrich-Alexander University Erlangen-Nürnberg, Universitätsstr. 22, 91504 Erlangen, Germany; 2 Institute of Pathology, University Hospital Erlangen, Friedrich-Alexander University Erlangen-Nürnberg, Krankenhausstr. 8-10, Erlangen, 91504 Germany; 3 Institute of Bioinformatics and Applied Biotechnology (IBAB), Bangalore, 560100 India; 4 Comprehensive Cancer Center Erlangen‑EMN (CCC ER‑EMN), Östliche Stadtmauerstr. 30, Erlangen, 91054 Germany

**Keywords:** ATR kinase, γ-H2AX, DNA repair, CAM model, DDR

## Abstract

**Background:**

The role of ATF2 in colon cancer (CC) is controversial. Recently, we reported that low ATF2 expression is characteristic of highly invasive tumors, suggesting that ATF2 might also be involved in therapy resistance. 5-Fluorouracil (5-FU) is the best-known chemotherapeutic drug for CC, but drug resistance affects its curative effect. To date, the role of ATF2 in the 5-FU response remains elusive.

**Methods/Results:**

For our study, we had available HCT116 cells (wild-type p53) and HT29 colon tumor cells (mutant p53) and their corresponding CRISPR‒Cas9-generated ATF2-KO clones. We observed that loss of ATF2 triggered dose- and time-dependent 5-FU resistance in HCT116 cells by activating the DNA damage response (DDR) pathway with high p-ATR^Thr1989^ and p-Chk1^Ser317^ levels accompanied by an increase in the DNA damage marker γ-H2AX in vitro and in vivo using the chicken chorioallantoic membrane (CAM) model. Chk1 inhibitor studies causally displayed the link between DDR and drug resistance. There were contradictory findings in HT29 ATF2-KO cells upon 5-FU exposure with low p-Chk1^Ser317^ levels, strong apoptosis induction, but no effects on DNA damage. In *ATF2*-silenced HCT116 p53^−/−^ cells, 5-FU did not activate the DDR pathway. Co-immunoprecipitation and proximity ligation assays revealed that upon 5-FU treatment, ATF2 binds to ATR to prevent Chk1 phosphorylation. Indeed, in silico modelling showed reduced ATR-Chk1 binding when ATF2 was docked into the complex.

**Conclusions:**

We demonstrated a novel ATF2 scaffold function involved in the DDR pathway. ATF2-negative cells are highly resistant due to effective ATR/Chk1 DNA damage repair. Mutant p53 seems to overwrite the tumor suppressor function of ATF2.

**Supplementary Information:**

The online version contains supplementary material available at 10.1186/s12885-023-10940-0.

## Introduction

ATF2, as a transcription factor of the leucine zipper family, behaves as a tumor suppressor or as an oncogene in a context- and stimulus-dependent manner [1]. When phosphorylated by mitogen-activated protein kinases (MAPKs), including ERK, SAPK/JNK, and p38 MAPK [2], it regulates the transcription of a plethora of genes, including those involved in apoptosis, cell growth, proliferation, and the DNA damage response (DDR). ATF2 functions as a homodimer or forms heterodimers with c-Jun, C/EBP, or Fos to activate its gene targets [3]. Low ATF2 expression is associated with worse prognosis in colon cancer (CC) patients and is characteristic of highly aggressive and invasive CC tumors [4].

To cope with DNA damage, phosphatidylinositol 3-kinase-related kinases (PIKKs), such as ATM, ATR, and DNA-PK, are recruited to DNA damage sites by several sensor protein complexes, such as the *Mre11–Rad50–Nbs1* complex (MRN) for ATM on DNA double-strand breaks (DSBs) or the ATR-ATRIP complex for ATR to respond to replication stress and single-stranded DNA damage [5, 6]. The signaling network after DDR involves the phosphorylation of various substrates of ATR and ATM, such as Chk1, Chk2, and p53 [7]*.* The DDR provides time for repair or triggers apoptosis in cases of extensive damage [8, 9]. Chk1, a serine/threonine kinase, is the major downstream target of ATR and can be phosphorylated at both the Ser317 and Ser345 sites. Chk1 controls cell cycle arrest at the S or G2 phase mainly by inducing Cdc25A/Cdc25C degradation [10, 11]. In the absence of Chk1, Cdc25A activates CDK2 by dephosphorylation of the Tyr15 site to promote unrestrained initiation of DNA replication during S phase. This leads to the stalling of replication forks with an accumulation of collapsed fork breaks, and cells switch to the G2–M checkpoint with damaged DNA. Moreover, loss of Chk1 fails to phosphorylate CDK1, and cyclin B1/CDK1 complex activity facilitates the transition from G2 to M phase. Ultimately, the cells enter mitosis with irreparable damage, resulting in cell death [12, 13].

The pyrimidine analog 5-fluorouracil (5-FU), which is widely used in the treatment of solid tumors, including CC [14], has been shown to induce genotoxic stress in tumor cells [15]. 5-FU-induced DDR is activated by the ATR/Chk1 axis, leading to cell cycle arrest in G2/M phase [10, 16]. Interestingly, lymphoma cells have been shown to be resistant to 5-FU by inhibition of S-phase progression in a Chk1-dependent manner [17]. Chk1 overexpression in CC has been linked to chemotherapy resistance [18]. p53-dependent Chk1 inhibition has been reported as a mechanism of apoptosis induction in CC [19]. Chk1 inhibitors are in clinical trials to sensitize CC cells to genotoxic stress and to overcome 5-FU resistance [20].

To our knowledge, the role of ATF2 in 5-FU resistance in CC cells has never been addressed so far. In this study, we report that the tumor suppressor ATF2 acts as a scaffold protein to reduce the binding efficiency between ATR and Chk1, thus preventing DDR and elevating 5-FU sensitivity in CC cells. In contrast, aggressive ATF2-negative cells are more resistant to 5-FU by promoting the ATR/Chk1-dependent DDR. We provide evidence that this novel function of ATF2 is p53 dependent.

## Materials and methods

### Cell lines and cell culture

The human colon cancer cell lines HCT116 (wild-type p53) and HT29 (mutant p53; R273H) were purchased from ATCC. Cells were maintained in RPMI-1640 (PAN Biotech P04-18,500) supplemented with 10% fetal bovine serum (FBS; PAN Biotech P30-3306) and 1% penicillin‒streptomycin (P/S; PAN Biotech P06-07,100). All cells were cultured at 37 °C with 5% CO_2_. CRISPR-Cas9 genome editing was used to stably knock out the *ATF2* gene in HCT116 (designated clones named E5 and F9) and HT29 (designated clones named B5 and F10) cells. The validation of the ATF2 knockout and functional description of the newly generated ATF2 knockout cell lines are given in Huebner et al. (2022) [4]. HCT116 p53^−/−^ cells were generously provided by Bert Vogelstein (Johns Hopkins) and maintained in McCoy’s 5A medium (Life Technologies, Darmstadt, Germany) containing 10% FBS and 1% P/S. The three cell lines were authenticated using Multiplex Cell Authentication by Multiplexion (Heidelberg, Germany). Mycoplasma-free status was verified.

### Drugs and chemicals

The autophagy inhibitor bafilomycin A1 (B1793-10 µg) was purchased from Sigma‒Aldrich and dissolved in 100% dimethyl sulfoxide (DMSO, Pan Biotech P6036720100) to a 16 μM stock solution; the Chk1 inhibitor (PF-477736, PZ0186) was purchased from Sigma‒Aldrich and dissolved in DMSO to a 10 mM stock solution; the JNK inhibitor (SP600125, tlrl-sp60) was purchased from InvivoGen and dissolved in DMSO to a 50 mM stock solution; and Z-VAD-FMK (S7023) was purchased from Selleckchem and dissolved in DMSO to a 10 mM stock solution. The single compound 5-fluorouracil (5-FU) was purchased from Sigma‒Aldrich and dissolved in DMSO to a 100 mM stock solution. All stocks were stored at − 20 °C.

### Western blotting

Western blotting analysis was performed as described previously [21]. Briefly, 20–60 μg protein was loaded into SDS‒PAGE gels and transferred onto 0.2 μm nitrocellulose membranes (GE Healthcare, 10,600,006) overnight. After blocking with 5% milk (Carl Roth, T145.2) in TBST for 1 h at room temperature, the membranes were incubated with corresponding primary antibodies overnight at 4 °C: caspase 9 (Cell Signaling, 9502, 1:1,000), Bcl-2 (Cell Signaling, 2872, 1:1,000), Bax (Cell Signaling, 5023, 1:1000), H2AX (Millipore, 07–627, 1:10,000 – 1:15,000), γ-H2AX (phospho-Ser139, Millipore, 05–636, 1:5,000 – 1:7,000), p-ATF2 E268 (p-Thr71, Abcam, ab32019, 1:5000), ATF2 E243 (Abcam, ab32160, 1:10,000), p-SAPK/JNK (p-Thr183/Tyr185, Cell Signaling, 4668, 1:1,000), SAPK/JNK (Cell Signaling, 9258, 1:1,000), p-p38 (p-Thr180/Tyr182, Cell Signaling, 9211, 1:1,000), p38 (Cell Signaling, 9212, 1:1,000), p-p44/42 (ERK1/2)(p-Thr202/Tyr204, Cell Signaling, 9101, 1:1,000), p44/42 (ERK1/2, Cell Signaling, 9102, 1:5,000), PARP (Cell Signaling, 9532, 1:1,000), p62 (Cell Signaling, 5114, 1:1,000), p-Chk1 (p-Ser317, Cell Signaling, 2344, 1:1,000), Chk1 (Santa Cruz, sc-8408, 1:500), p-ATR (p-Thr1989, Cell Signaling, 30,632, 1:1,000), ATR (Cell Signaling, 13,934, 1:1,000), and secondary antibodies (anti-mouse and anti-rabbit IgG peroxidase conjugated, Pierce, Rockford, IL, USA, 1:10,000), GAPDH-HRP (Abnova, MAB5476, 1:50,000–1:100,000). Experiments were performed in independent biological duplicates. For Western blot quantification of -H2AX/H2AX, p-Chk1^Ser317^/Chk1, and p-ATR^Thr1989^/ATR, band intensities were quantified by ImageJ (National Institute of Health, USA) and normalized to each corresponding GAPDH (housekeeper). Ratios were calculated by dividing phosphorylated protein of interest by nonphosphorylated protein of interest. Ratios for cleaved PARP were determined by ImageJ, but ratios were calculated by dividing cleaved PARP by noncleaved PARP.

### Co-Immunoprecipitation (Co-IP)

Co-IP was performed according to the ‘Dynabeads® Protein G Immunoprecipitation Kit’ manual (Thermo Fisher, 10007D) with slight adjustments. Briefly, protein lysate (500 μg/sample) was adjusted to a final volume of 200 μl and incubated with primary antibodies (ATF2 E243 (Abcam, ab32160, 1:50), p53 (Santa Cruz, sc-126, 1:20), Chk1 (Santa Cruz, sc-8408, 1:20)) overnight on rotation at 4 °C. The next day, the mixture was incubated with 30 µl Dynabeads® for 15 min at RT, which had been prewashed once with 200 µl Ab-Binding & Washing Buffer. Immunocomplex was then washed with 200 µl Washing Buffer 3 times. The Dynabeads®-Ab-antigen complex was resuspended in 100 µl Washing Buffer and transferred to new Eppendorf tubes. The supernatant was removed, 20 µl elution buffer and 4 µl SDS (6X) loading buffer were added, and the samples were subjected to SDS‒PAGE. Western blotting was performed following standard procedures.

### Chorioallantoic membrane (CAM) assay and immunohistochemistry

Fertilized specific-pathogen-free eggs (Valo Biomedia) were incubated at a temperature of 37 °C and constant air humidity of 70%. Eggs were opened on day 8 of chicken embryo development, and the hole was sealed with surgical tape. On day 9, 1.0 × 10^6^ cells pretreated with 15 μM 5-FU for 48 h were resuspended in a mixture of 20 µl RPMI 1640 medium and 20 µl Matrigel. The 40 µl cell pellet was slowly placed onto the CAM. After incubating for 5 days, xenografts with surrounding CAM were harvested and fixed in 4% paraformaldehyde for 24 h before paraffin embedding. Serial slides were stained with H&E and antibodies against ATF2 (E243, Abcam 1:100,000), Ki67 (Dako, 1:100), pan cytokeratin (Zytomed, 1:40), p-Chk1^Ser317^ (Abcam, 1:2,000), and γ-H2AX (Abcam, 1:2,000).

### RNA Interference

Interfering ON-TARGETplus SMARTpool ATF2 siRNA (D-009871–00-0005) and scramble control siRNA (ON-TARGETplus Nontargeting pool, D-001810–10-05) were purchased from Dharmacon. Transfection was performed in 6-well plates using Lipofectamine RNAiMAX reagent (Thermo Fisher, 13,778,075) in OptiMEM (Thermo Fisher, 31,985,062) containing 25 pmol of siRNA/scramble in each well. Cells were counted and seeded again after 48 h of transfection at 37 °C and 5% CO, followed by 48 h of treatment with 5-FU. Knockdown efficiency was assessed by Western blotting.

### Colony formation assay

Cells were pretreated with 15 µM 5-FU or DMSO. After 48 h of incubation, the cells were trypsinized, and 1000 cells were seeded in a 6 cm plate with normal growth medium and incubated for an additional 10 days (control group) and 20 days (treatment group). As the colonies became visible, the cells were fixed and permeabilized with 70% methanol for 20 min. Afterwards, colonies were stained with crystal violet for 20 min, washed with water and allowed to dry. Pictures were taken with a digital camera, and the number of colonies was recognized by Image-Pro Plus software.

### Annexin-propidium iodide apoptosis assay

Detection of apoptosis was performed by Annexin-PI staining as described previously [22]. Cells were treated with 15 μM 5-FU for 48 h or with normal medium for 24 h. The fluorescent signal was measured by flow cytometry (BD FACSCanto® II, BD Biosciences). The data were evaluated using FlowJo 7.6.5 software.

### Immunofluorescence

Cells were seeded on coverslips and treated with 15 μM 5-FU or DMSO for 48 h. After the treatment, the cells were fixed with 4% paraformaldehyde for 20 min and permeabilized with 0.1% Triton X-100 in PBS for 10 min. Afterwards, the cells were blocked with 3% BSA in PBS for 30 min on a shaker and subsequently incubated with primary antibody (p-ATF2 E268 (p-Thr71, Abcam, ab32019, 1:500)), which was diluted in 3% BSA in PBS for 1 h at room temperature, and secondary antibody (goat anti-rabbit IgG, Alexa Fluor 555, Thermo Fisher, A-21428, 1:500), which was diluted in 1% BSA for 1 h in a dark room. Nuclei were stained with DAPI (Sigma‒Aldrich, MBD0015-5ML, 1:1,000) in PBS for 25 min. Fluorescence images were taken by a Nikon Ti-S fluorescence microscope.

### Duolink® Proximity Ligation Assay (PLA)

Cells were seeded onto an Ibidi µ-Slide chamber. After 48 h of treatment with 5-FU or DMSO, cells were fixed with 4% paraformaldehyde for 20 min and permeabilized with 0.1% Triton-X for 15 min. Thereafter, the cells were blocked with 3% BSA for 30 min and incubated with a mixture of primary antibodies for 1 h (p-ATR (p-Thr1989, Thermo Fisher, MA5-27,731, 1:500) and ATF2 E243 (Abcam, ab32160, 1:500)) diluted in 3% BSA. Then, the cells were incubated with secondary antibody for 1 h in a humidity chamber at 37℃ (mixture of Duolink® In Situ PLA® Probe Anti-Mouse MINUS (Sigma‒Aldrich, DUO92001-30RXN) and Duolink® In Situ PLA® Probe Anti-Rabbit MINUS (Sigma‒Aldrich, DUO92005-30RXN)). Hybridization, ligation, amplification, and detection were performed according to the manufacturer’s instructions (Duolink™ In Situ Detection Reagents Red, Sigma‒Aldrich, DUO92008). Finally, cells were stained with DAPI (1:1,000) in 0.01 × Wash Buffer B for 10 min before acquiring images. The technical negative control was incubated with PLA secondary antibody; the positive control was incubated with a mixture of p21/p-Chk2^Thr68^ primary antibodies (p21 (Cell Signaling, 2946, 1:400) and p-Chk2 (p-Thr68, Cell Signaling, 2197, 1:200)). PLA foci were evaluated by the software BlobFinder [23]. Nuclei and PLA foci were automatically detected by the software to allow quantification of PLA foci (nucleus and cytoplasm) per cell. Signals detected outside of cells were categorized as background and served for background correction.

### Modelling of the ATR-Chk1 complex

Energy-minimized models of ATR, ATF2 and Chk1 were used to develop the complexes. The ATR-ATF2 complex was generated by docking individual protein structures using the protein‒protein docking server ClusPro [24] (https://cluspro.org/home.php). The cluster scores of the complexes from ClusPro were utilized to select the most reliable ATR-ATF2 complex. Furthermore, the interactions (hydrogen bonds) between the proteins were calculated using the Protein Interaction Calculator [25]. Similarly, the ATR-Chk1 complex was also developed using the ClusPro server, and the interaction was calculated using the Protein Interaction Calculator.

### Modelling of the (ATR-ATF2)-Chk1 complex

This was further used to dock the ATR-ATF2 complex with Chk1 to develop the ATR-ATF2-Chk1 complex. Energy of the developed models and complexes computed using the Gromos96 force field [26]. Further active site pockets of ATR and ATF2 were predicted using the CASTp online server [27]. The interacting active site residue region of ATR and ATF2 was knocked out from the complex using Pymol [28], further protein‒protein docking was carried out using the ClusPro server, and interactions were calculated.

## Results

### 5-FU induces ATF2-dependent apoptosis in HCT116 tumor cells

**Fig. 1:**
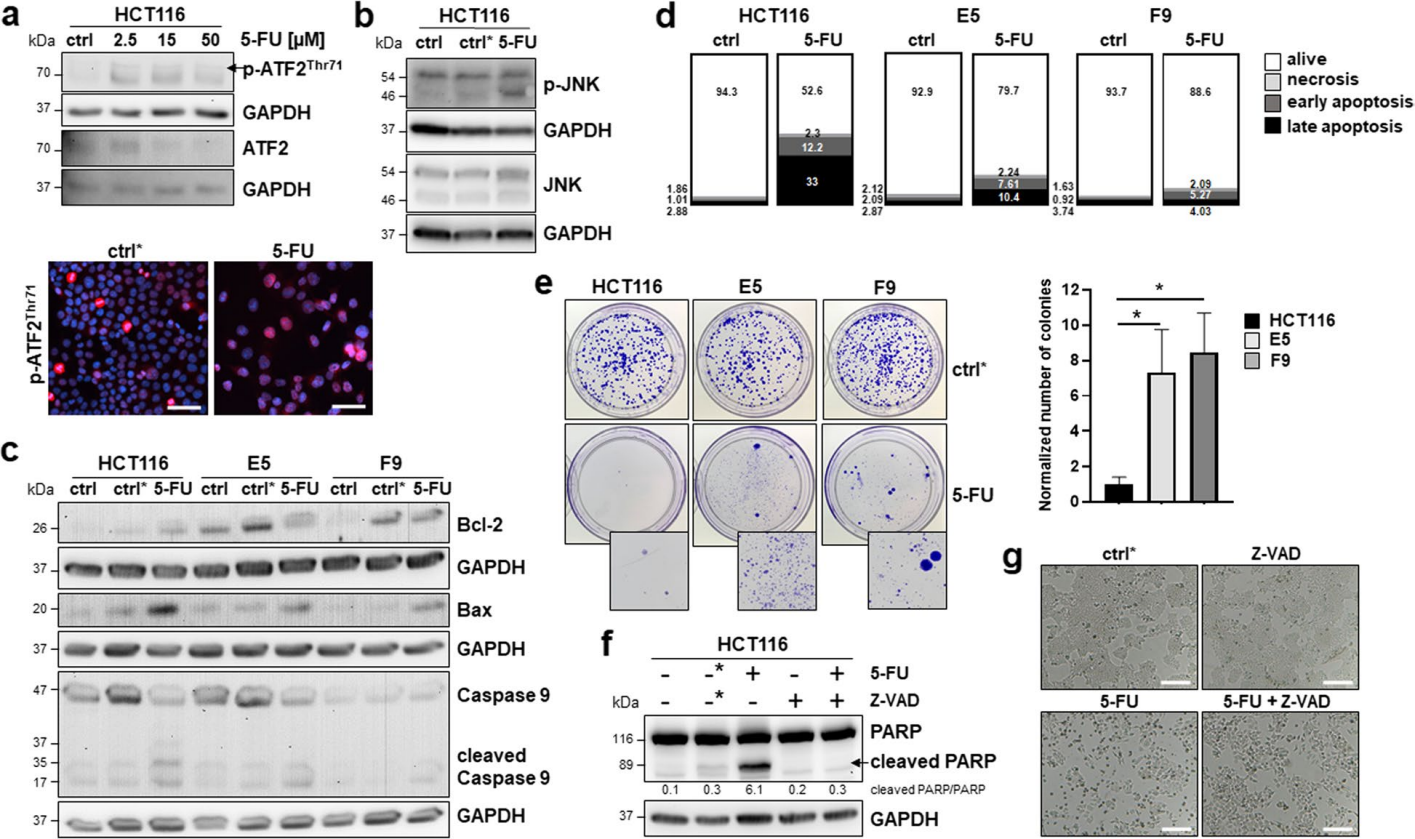
ATF2-KO cells are more resistant to 5-FU therapy. **a** 5-FU treatment at different doses (2.5 µM, 15 µM, and 50 µM) induced p-ATF2^Thr71^ and ATF2 in Western blotting. ctrl: 24 h nontreated cells for control. For immunofluorescence, the cells were seeded on coverslips and then treated with 15 µM 5-FU for 48 h, and p-ATF2^Thr71^ was visualized by Alexa Fluor 555 goat anti-rabbit staining (red). DAPI was used for cell nuclei (blue). 20X magnification images were taken with fluorescence microscopy, scale bar: 50 μm; ctrl*: 48 h DMSO treated cells for control. **b** 5-FU (15 µM for 48 h) induces p-JNK and JNK in Western blotting; ctrl: 24 h nontreated cells for control, ctrl*: 48 h DMSO treated cells for control. **c** Representative Western blotting for intrinsic apoptotic markers Bcl-2, Bax, and caspase 9, cleaved caspase 9 in HCT116, and ATF2-KO E5 and F9 cells upon 15 µM 5-FU exposure for 48 h; ctrl: 24 h nontreated cells for control, ctrl*: 48 h DMSO treated cells for control, GAPDH was used as loading control. **d** Flow cytometry analysis of Annexin-PI staining in HCT116 and ATF2-KO E5 and F9 cells upon 15 µM 5-FU exposure for 48 h; percentages of alive, early apoptotic, late apoptotic and necrotic populations are given, ctrl: 24 h nontreated cells for control. **e** Images showing the effects of 5-FU on long-term cell viability by colony formation assay. Digitally enlarged images of 5-FU-treated plates show the presence of cells in each condition. Cells were treated for 48 h with 15 µM 5-FU or DMSO (ctrl*), t test (n = 4) *P ≤ 0.05. The normalized number of colonies was calculated against the corresponding DMSO control. Ratios for E5 and F9 were normalized against the HCT116 ratio. **f** Western blotting for PARP, cleaved PARP and morphologic changes upon treatment. Ratios were calculated as cleaved PARP versus noncleaved PARP (ImageJ). For visual reasons, the ratios were multiplied by 10. **g**, Cells were pretreated with 1 h Z-VAD (50 μM), followed by treatment with 15 μM 5-FU for 48 h; 24 h nontreated cells were used as controls (-), 48 h DMSO-treated cells were used as controls (-*), and GAPDH was used as a loading control

Initially, we examined whether ATF2 was activated after 5-FU treatment. The IC50 level of 5-FU in HCT116 cells obtained from previous studies was 15 µM [29], and we observed an upregulation of p-ATF2^Thr71^ levels accompanied by a downregulation of ATF2 after 48 h of treatment with 2.5 µM, 15 µM and 50 µM 5-FU (Fig. 1a). This increase in p-ATF2^Thr71^-positive cells was confirmed by immunofluorescence of cells treated with 15 µM 5-FU (Fig. 1a). Next, we aimed to identify which of the upstream MAP kinases JNK, ERK, or p38 was targeted by 5-FU. We performed Western blotting for activated and total forms of these three MAPKs and showed that only p-JNK was upregulated after 15 µM 5-FU treatment in HCT116 cells for 48 h, suggesting JNK-dependent ATF2 signaling (Fig. 1b, Suppl. Figure S1a). To examine potential ATF2-dependent effects upon 5-FU exposure, HCT116 and CRISPR‒Cas9-generated ATF2 knockout (HCT116 ATF2-KO) clones (named E5 and F9) were treated for 48 h with 15 µM 5-FU. When examining the levels of pro-apoptotic Bax and anti-apoptotic Bcl-2 proteins, we verified an ATF2-dependent induction of the intrinsic apoptotic pathway (Fig. 1c). HCT116 ATF2-KO clones were resistant to 5-FU, showing the highest Bcl-2 and lowest Bax protein levels. Accordingly, HCT116 cells had the highest levels of cleaved caspase 9 and apoptotic cell fractions in Annexin-PI staining (Fig. 1c, d).

Additionally, resistance to 5-FU was visible in the colony formation assay, in which 5-FU-treated HCT116 ATF2-KO clones had a higher capability to grow as colonies than 5-FU-treated HCT116 cells (Fig. 1e). Pretreatment of HCT116 cells with 50 µM of the pancaspase inhibitor Z-VAD-FMK for 1 h prior to 5-FU treatment showed complete inhibition of apoptosis, as seen in Western blotting by cleaved PARP, a well-known substrate of effector caspase 3 (Fig. 1f), and in morphology (Fig. 1g, Suppl. Figure S1b). Interestingly, treatment with the autophagy inhibitor bafilomycin A1 showed that autophagy seemed to not be responsible for the apoptosis resistance of HCT116 ATF2-KO clones since inhibition of autophagy did not result in significantly elevated PARP cleavage (Suppl. Figure S1c) Finally, these findings suggest that HCT116 ATF2-KO clones are less sensitive to the 5-FU-induced caspase-dependent apoptotic pathway.

### 5-FU treatment induces an ATF2-dependent DDR pathway

**Fig. 2:**
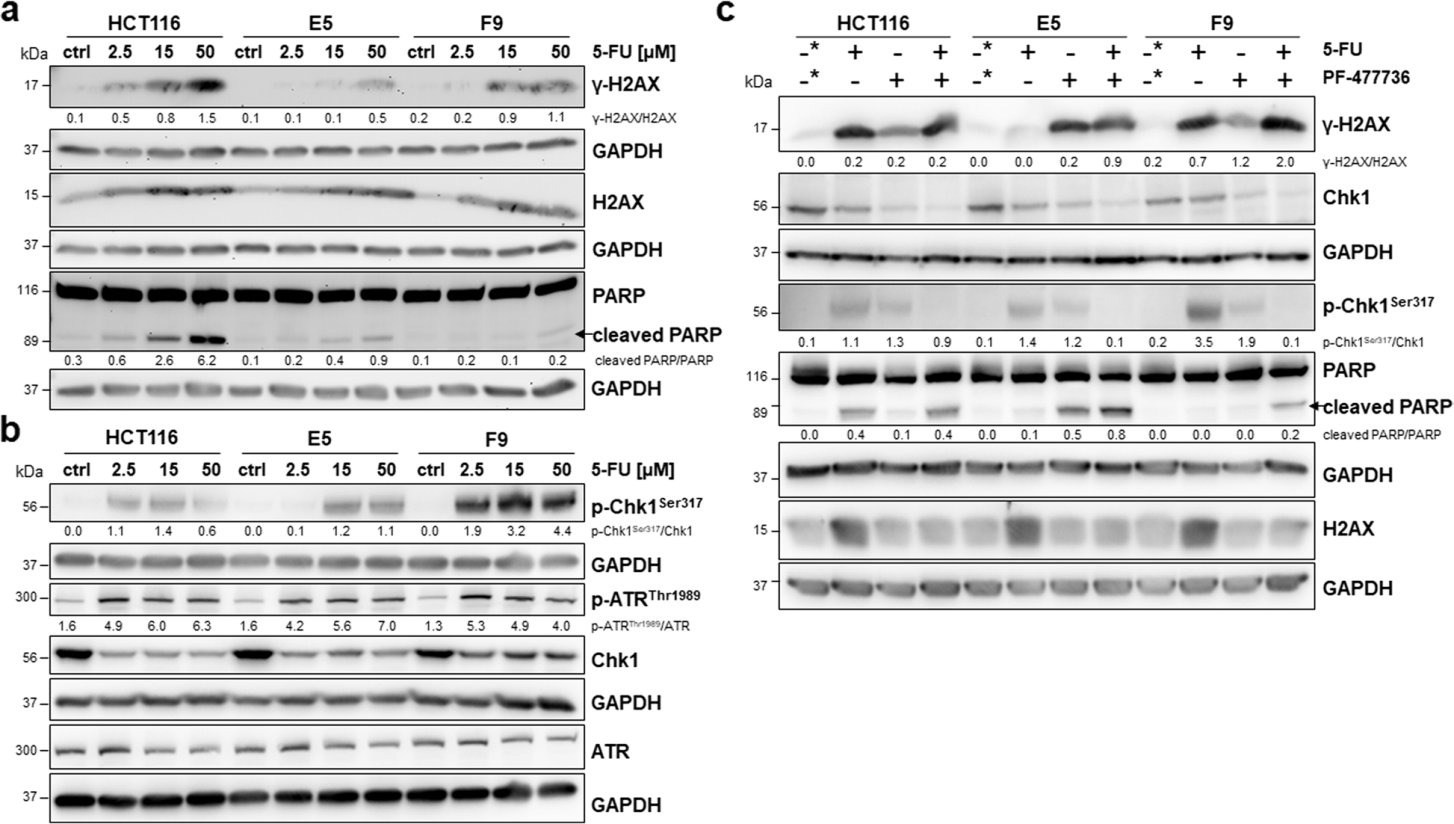
Activation of the ATR/Chk1 pathway plays an important role in ATF2-mediated 5-FU resistance. **a** Representative Western blotting for DNA damage marker γ-H2AX, total H2AX and apoptosis marker PARP after 5-FU treatment of HCT116 and HCT116 ATF2-KO clones E5 and F9 with different doses (2.5 µM, 15 µM, and 50 µM); ctrl: 24 h nontreated cells for control, GAPDH was used as loading control. Band intensities were quantified using ImageJ analysis software, and ratios were calculated against the GAPDH band intensity. For cleaved PARP, given ratios were calculated as cleaved PARP versus noncleaved PARP (ImageJ) and multiplied by 10 for visual reasons. **b** Representative Western blotting for DDR markers p-ATR^Thr1989^, total ATR, p-Chk1.^Ser317^, and total Chk1 after 5-FU treatment of HCT116 and HCT116 ATF2-KO clones E5 and F9 with different doses (2.5 µM, 15 µM, and 50 µM); ctrl: 24 h nontreated cells for control, GAPDH was used as loading control. Band intensities were quantified using ImageJ analysis software, and ratios were calculated against the GAPDH band intensity. **c** Representative Western blotting after pretreating HCT116 and HCT116 ATF2-KO clones E5 and F9 with the Chk1 inhibitor PF-00477736 (1.65 nM) for 1 h followed by 5-FU (15 µM) for 48 h; 48 h DMSO-treated cells were used as control (-*), GAPDH was used as loading control. Band intensities were quantified using ImageJ analysis software, and ratios were calculated against the GAPDH band intensity. For cleaved PARP, given ratios were calculated as cleaved PARP versus noncleaved PARP (ImageJ)

To identify whether the DDR was responsible for 5-FU resistance in HCT116 ATF2-KO clones, we treated HCT116 cells and their ATF2-KO clones with 15 µM 5-FU and detected the expression levels of the DNA damage marker γ-H2AX/H2AX, the damage sensor p-ATR^Thr1989^/ATR and its major target p-Chk1^Ser317^/Chk1 by Western blotting. There was lower DNA damage detectable in HCT116 ATF2-KO clones accompanied by higher levels of p-Chk1^Ser317^ (Fig. 2a, b), whereas the p-ATR^Thr1989^/ATR ratio did not differ in an ATF2-dependent manner. The activated DDR was reflected by lower PARP cleavage in the ATF2-KO clones (Fig. 2a). Since 5-FU leads to both DNA breaks and stalled replication forks, we investigated ATR/Chk1, the pathway that predominantly repairs damage associated with replication forks. We examined Chk1 activation by measuring the phosphorylation at residues Ser317 of Chk1 and Thr1989 ATR for ATR activation. Consistent results upon 5-FU treatment were found when we reduced ATF2 expression levels in HCT116 cells by JNK inhibitor treatment (Suppl. Figure S1d).

If the increase in p-Chk1^Ser317^ levels mediated more effective DNA repair, Chk1 inhibition should result in apoptosis induction in HCT116 ATF2-KO clones. We successfully inhibited Chk1 when treating the cells with 1.65 nM of the inhibitor PF-477736 [30] for 1 h prior to 5-FU exposure and showed that the p-Chk1^Ser317^ signals nearly disappeared in Western blotting (Fig. 2c). The loss of p-Chk1^Ser317^ was accompanied by a remarkable increase in γ-H2AX levels and elevated cleaved PARP band signals in HCT116 ATF2-KO clones, whereas there was lower apoptosis induction in HCT116 cells (Fig. 2c).

### ATF2-KO exacerbates resistance to apoptosis via Chk1 activation in vivo

**Fig. 3:**
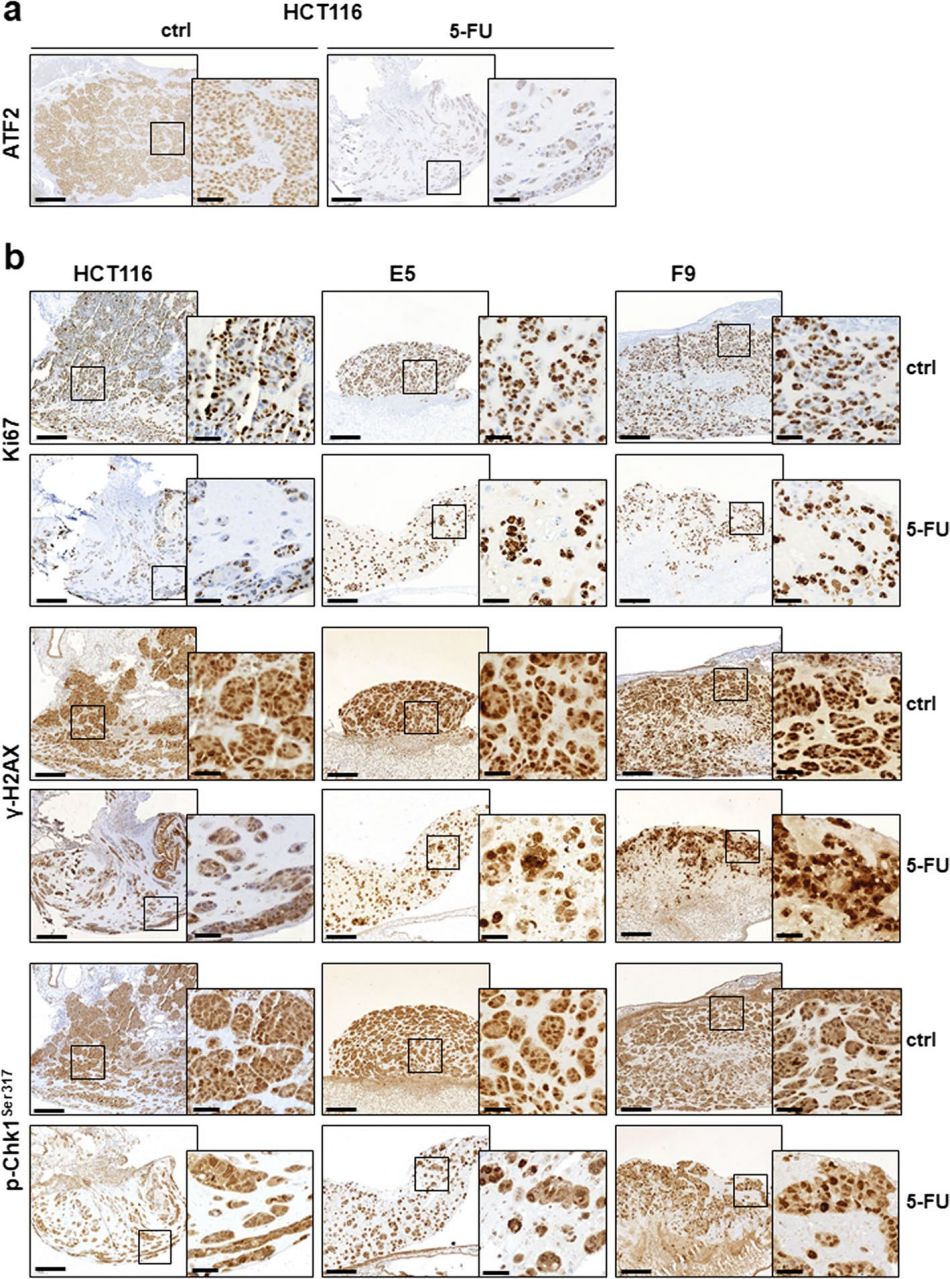
ATF2 loss induces a resistant pattern in CAM xenografts. The immunohistochemistry panel shows (**a**) ATF2 and (**b**) Ki67, γ-H2AX, and p-Chk1^Ser317^ staining of formalin-fixed paraffin-embedded (FFPE) tumor tissues harvested from the CAM. The tumors formed by 120 h inoculation with 5-FU or normal medium (ctrl) as a control in HCT116, E5, and F9 cells. The scanned images of CAM sections were taken at 10X magnification (overview sections, scale bar: 200 μm) and 50X magnification (black box circled the detailed area; scale bar: 40 μm)

To confirm ATF2-mediated resistance to 5-FU in vivo, we analysed xenografts of our HCT116 cell line set in the chorioallantoic membrane (CAM) model. Cells were treated with 5-FU (15 µM) for 48 h, supernatants containing the dead cells were discarded, and the adherent (potentially resistant) cell population was transplanted onto the CAM of a 9-day-old chicken embryo. First, we observed that xenografts derived from 5-FU-treated HCT116 cells displayed lower ATF2 expression than untreated xenografts (Fig. 3a, Suppl. Figure S2). The apoptosis resistance to 5-FU in xenografts derived from HCT116 ATF2-KO clones was reflected by a higher number of proliferating cells measured by Ki67 immunostaining (Fig. 3b, Suppl. Figure S2). HCT116 cells did not efficiently activate the DDR, since only a few cell nuclei were positive for p-Chk1^Ser317^. Similar to the in vitro observations, ATF2-KO xenografts showed higher levels of p-Chk1^Ser317^ than HCT116 cells (Fig. 3b, Suppl. Figure S2). Unexpectedly, γ-H2AX levels were also higher in ATF2-KO xenografts, suggesting that the activated DDR in vivo led to an accumulation of further damage in the cells, but it was well tolerated by these resistant surviving cells (Fig. 3b, Suppl. Figure S2). When identifying epithelial tumor cells by pan cytokeratin staining and growth pattern by H&E staining, HCT116 consisted of a dense tumor mass, while ATF2 KO clones E5 and F9 grew as loosely packed tumors (Suppl. Figure S3). After 5-FU treatment, the number of tumor cells in HCT116-derived xenografts was significantly reduced with large areas of tumor-free Matrigel, while HCT116 ATF2 KO tumors had a higher cell density, reflecting more surviving cells (Suppl. Figure S3).

### 5-FU activates an ATF2/p-ATR^Thr1989^ complex

**Fig. 4:**
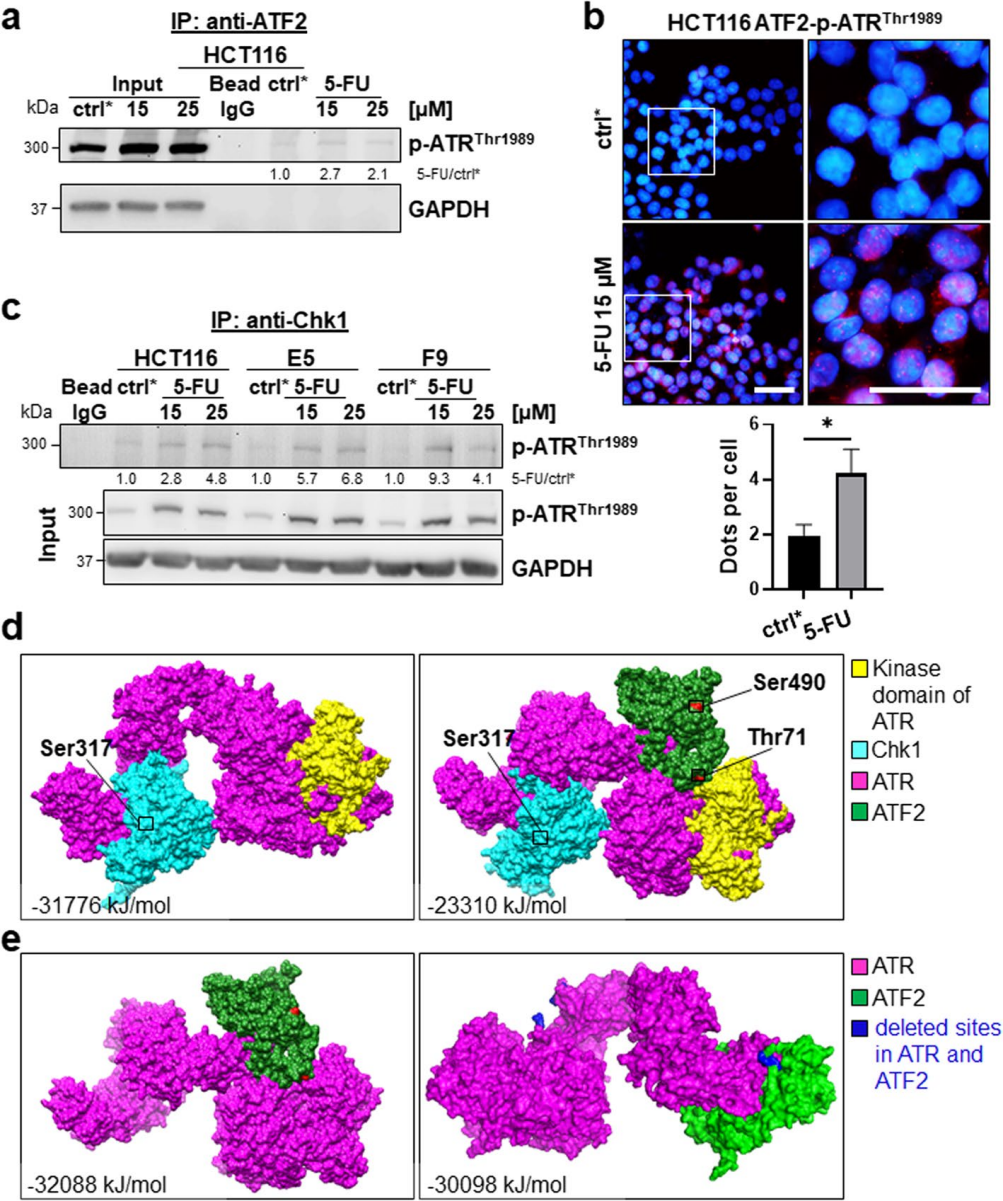
Interaction between ATF2 and ATR. **a** Co-IP performed with an anti-ATF2 antibody in HCT116 cells. The Co-IP or whole cell extract (Input) was subjected to SDS–PAGE followed by Western blotting; ctrl*: 48 h DMSO treated cells, GAPDH was used as loading control for input. Band intensities were quantified using ImageJ analysis software, and ratios were calculated against the corresponding ctrl* band intensity. **b** The ATF2-p-ATR^Thr1989^ complex was analysed by proximity ligation assay (PLA) on a fluorescence microscope. The interaction between ATF2 and p-ATR.^Thr1989^ was visualized by red PLA dots. Nuclei were counterstained with DAPI. Merged images were taken at 40X magnification, scale bar: 50 μm; right: digitally enlarged images, scale bar: 50 μm. Brightness and contrast were adjusted evenly for all conditions. **c** Co-IP performed with an anti-Chk1 antibody in HCT116, E5, and F9 cells. The Co-IP or whole cell extract (Input) was subjected to SDS–PAGE followed by Western blotting; ctrl*: 48 h DMSO-treated cells. GAPDH was used as a loading control for input. Band intensities were quantified using ImageJ analysis software, and ratios were calculated against the corresponding ctrl* band intensity. **d** Modelling of ATF2 and Chk1 was obtained by homology structural modelling using MODELLER v10.0: ATF2 (green) was modelled using two X-ray crystallographic structures, 6ZQS_chainB and 1T2K_chainD, and one NMR structure, 1BHI, as templates; Chk1 protein (cyan) was modelled using two X-ray crystallographic structures, 2X8D and 5WI2_A, as templates; the remaining portions were predicted using RaptorX. The modelled structures of ATF2 and Chk1 were subjected to energy minimization using GROMACS. Based on the lowest energy values and fewest Ramachandran outliers obtained from ProCheck, the final modelled structures of ATF2 and Chk1 were chosen. ATR (purple) was derived from the complete cryo-EM structure of the human ATR-ATRIP complex (PDB: 5YZ0, resolution 4.70 A). ATR kinase domain (yellow). The ATR-Chk1 complex was generated by docking individual protein structures using the protein‒protein docking server ClusPro. Cluster scores of the complexes from ClusPro were utilized to select the ATR-Chk1 complex. This complex was further used to dock the ATR-Chk1 complex with ATF2. Finally, the protein complexes were analysed using the PDBePISA (Proteins, Interfaces, Structures and Assemblies) tool to determine the binding energy and dissociation pattern of the docked complex. **e** Modelling of the ATR and ATF2 interaction upon deletion of the binding sites. Active site pockets of ATR and ATF2 were predicted using the CASTp online server [27]. The interacting active site residue region of ATR and ATF2 was knocked out from the complex using Pymol [28], further protein‒protein docking was carried out using the ClusPro server, and interactions were calculated. The energies of the developed models and complexes were computed using the Gromos96 force field [26]

Next, we aimed to understand how ATF2 affected the ATR/Chk1 axis. We found an interaction of ATF2 and p-ATR^Thr1989^ (activated ATR) in co-immunoprecipitation (Co-IP) in HCT116 cells; this complex was newly formed upon 5-FU treatment (Fig. 4a). This finding was confirmed by proximity ligation assay (Fig. 4b, Suppl. Figure S4). Additionally, we identified more complexes composed of Chk1 and p-ATR^Thr1989^ in HCT116 ATF2-KO clones by Co-IP, which could explain our findings for p-Chk1^ser317^ in Western blotting (Fig. 4c). [31]. In silico modelling allows us to understand how proteins interact. Protein‒protein docking revealed that in wild-type (WT) HCT116 cells, when both ATF2 and Chk1 are present, both of these proteins interact with ATR. We found that the ATR-Chk1 complex stabilized at a comparatively higher energy level, suggesting that the binding of ATF2 with ATR (ΔiG = -32,088 kJ/mol) is stronger than the binding of Chk1 with ATR (ΔiG = -31,776 kJ/mol). The complex of ATF2, Chk1 and ATR shows higher energy (ΔiG = -23,310 kJ/mol), suggesting an unstable triple complex (Fig. 4d). In the absence of ATF2, Chk1 is observed to interact more efficiently with ATR, which leads to high phosphorylation levels of Chk1 at the Ser317 position. This can be observed clearly in the docked complex shown in Fig. 4d. ATF2 seems to directly interact with the kinase domain of ATR, as shown in Fig. 4e. In contrast, Chk1 seems to bind far away from the kinase domain (Fig. 4d). The dissociation pattern predicted by the PDBe PISA tool also suggests an alignment with the results obtained in the Co-IP study.

### Mutant p53 affects ATF2-mediated suppression of the DDR response to 5-FU

**Fig. 5:**
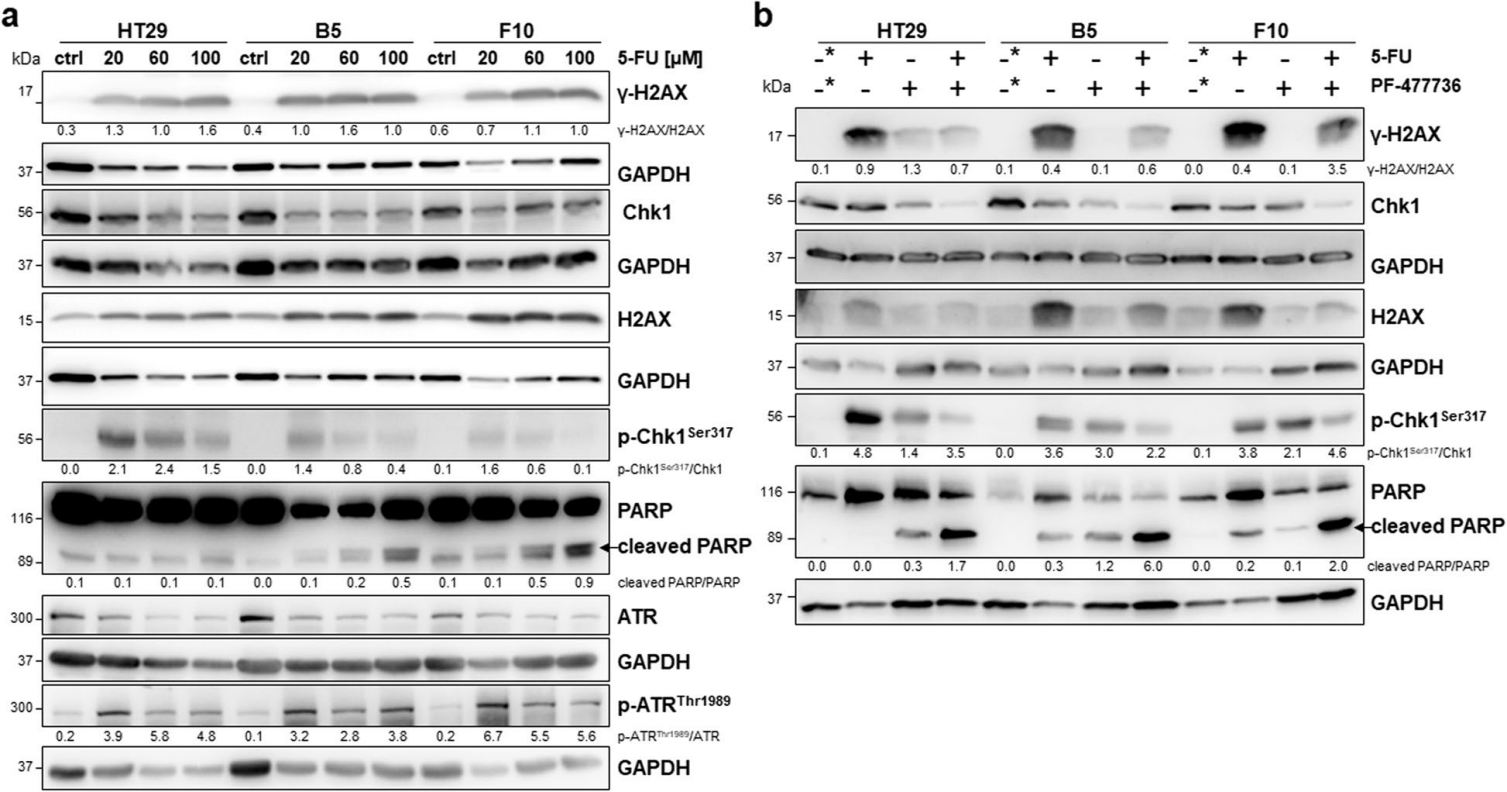
**a** Representative Western blotting analysis of dose-dependent 5-FU-induced γ-H2AX, H2AX, PARP, cleaved PARP, p-Chk1^Ser317^, Chk1, p-ATR.^Thr1989^, ATR in HT29, B5, and F10 cells; ctrl: 24 h nontreated cells for control, GAPDH was used as loading control. Band intensities were quantified using ImageJ analysis software, and ratios were calculated against the GAPDH band intensity. For cleaved PARP, given ratios were calculated as cleaved PARP versus noncleaved PARP (ImageJ). **b** Western blotting analysis was performed by pretreating cells with the Chk1 inhibitor PF-00477736 (1.65 nM) for 1 h followed by 5-FU (15 µM) for 48 h; 48 h DMSO-treated cells were used as a control (-*), and GAPDH was used as a loading control. Band intensities were quantified using ImageJ analysis software, and ratios were calculated against the GAPDH band intensity. For cleaved PARP, given ratios were calculated as cleaved PARP versus noncleaved PARP (ImageJ)

To verify our findings, we treated another colon cancer cell line, HT29, which is mutant for p53, with 5-FU. Since HT29 cells are well known for being less sensitive to 5-FU than HCT116 cells, we used higher doses of 5-FU (20 µM, 60 µM, 100 µM) and performed Western blotting for all markers of the DDR in HT29 cells and their two ATF2-KO clones B5 and F10 (Fig. 5a). Although the IC50 for 5-FU has been reported to be 60 µM for 48 h [21], we already observed effects on p-Chk1^Ser317^ and p-ATR^Thr1989^ levels at lower doses comparable to those used for HCT116 cells. Interestingly, p-ATR^Thr1989^ protein expression was induced upon 5-FU in all three cell lines, whereas the p-Chk1^Ser317^ levels were induced at a lower level with the tendency to be reduced or not detectable at the highest dose of 100 µM in ATF2-KO clones. Cleaved PARP levels increased in a dose-dependent manner, suggesting that apoptosis induction at high 5-FU doses was associated with the lowest p-Chk1^Ser317^ levels but was independent of DNA damage (Fig. 5a).

Next, we aimed to examine to what extent the DDR response upon 5-FU treatment is dependent on Chk1 in p53-mutant cells and their ATF2-KO clones. First, we verified a decrease in the expression of both phosphorylated p-Chk1^Ser317^ and total Chk1 levels after treatment with the Chk1 inhibitor PF-477736, indicating effective Chk1 kinase inhibition (Fig. 5b). When treating cells with a combination of 5-FU and Chk1 inhibitor, the sensing by the upstream p-ATR should be the same for all three cell lines. Indeed, the γ-H2AX levels did not change in all three cell lines when comparing 5-FU with combination treatment. Next, we would expect that the inhibition of Chk1 in HT29 cells should increase apoptosis upon 5-FU treatment, as demonstrated in Chk1-addicted HCT116 ATF2-KO clones. Since we reached rather similar levels of p-Chk1^Ser317^ after combination treatment, the apoptosis levels increased more than tenfold in all three cell lines (Fig. 5b)*.* In summary, in cells with defective p53, the inhibition of Chk1 function resulted in massive cell death in all three HT29 cell lines (Fig. 5b).

**Fig. 6:**
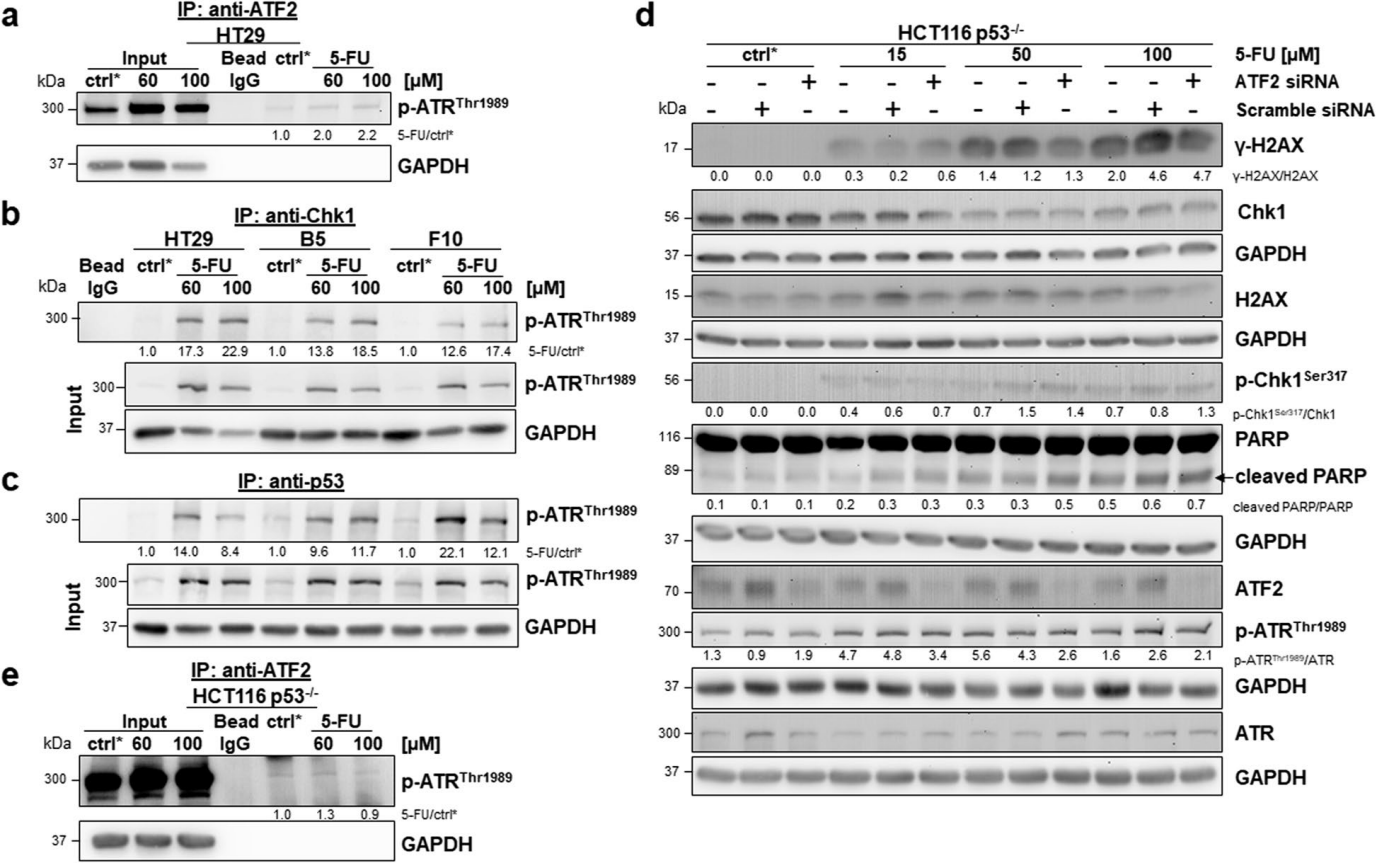
No ATF2-dependent effect on DDR in HCT116 p53^-/-^ cells upon 5-FU treatment. **a**, **b**, **c** Co-IP performed with an anti-ATF2 antibody in HT29 cells (a) or with an anti-Chk1 antibody (b) or with an anti-p53 antibody (c) in HT29, B5, and F10 cells. The Co-IP or whole cell extract (Input) was subjected to SDS–PAGE followed by Western blotting; ctrl*: 48 h DMSO treated cells for control, GAPDH was used as loading control for input. Band intensities were quantified using ImageJ analysis software, and ratios were calculated against the corresponding ctrl* band intensity. **d** *ATF2* silencing through RNAi-mediated *ATF2* knockdown. Representative Western blotting for ATF2, γ-H2AX, H2AX, PARP, cleaved PARP and p-Chk1^Ser317^, Chk1, p-ATR^Thr1989^, ATR after treatment with various doses of 5-FU for 48 h; ctrl*: 48 h DMSO treated cells for control, GAPDH was used as loading control. Band intensities were quantified using ImageJ analysis software, and ratios were calculated against the GAPDH band intensity. For cleaved PARP, given ratios were calculated as cleaved PARP versus noncleaved PARP (ImageJ). **e** Co-IP performed with an anti-ATF2 antibody in HCT116 p53-/- cells. The Co-IP or whole cell extract (Input) was subjected to SDS–PAGE followed by Western blotting; ctrl*: 48 h DMSO treated cells for control, GAPDH was used as loading control for input. Band intensities were quantified using ImageJ analysis software, and ratios were calculated against the corresponding ctrl* band intensity

Subsequently, we wondered what might have caused the opposite effect on p-Chk1^Ser317^ expression upon 5-FU treatment, which was low in HCT116 cells and high in HT29 cells. As described for p53-WT HCT116 cells, under 5-FU, the complex between ATF2 and p-ATR^Thr1989^ is also triggered in HT29 cells, as demonstrated by Co-IP (Fig. 6a). Correspondingly, a p-ATR^Thr1989^-Chk1 interaction was verified by Co-IP. This complex was newly created after treatment with 60 µM 5-FU for 48 h in HT29 cells and to a lesser extent in HT29 ATF2-KO clones (Fig. 6b). Moreover, we proved that the complex formed by mutant p53 and p-ATR^Thr1989^ was stronger in HT29 ATF2-KO clones, especially at higher doses (Fig. 6c). Wild-type p53 and p-ATR^Thr1989^ formed a complex in HCT116 cells and ATF2-KO clones upon 5-FU exposure (Suppl. Figure S5a). When investigating the p53-ATF2 complex by proximity ligation assay, we observed that in HCT116 control cells, such a complex did not exist, but it was newly formed upon 5-FU exposure (Suppl. Figure S5b). In contrast, in HT29 control cells, a strong ATF2-p53 complex already existed, whereas upon 5-FU exposure, ATF2 seemed to be released from the p53-ATR complex (Suppl. Figure S5c, Suppl. Figure S6). Obviously, mutant p53 was able to interfere with ATR-mediated Chk1 phosphorylation by stronger binding to ATR when ATF2 was lost. This might explain the higher sensitivity of HT29 ATF2-KO clones to 5-FU treatment.

### Influence of ATF2 on the ATR/Chk1 pathway is p53-dependent

Due to controversial results in HCT116 cells with wild-type p53 and HT29 cells with mutant p53, we used an HCT116 p53-knockout cell line (HCT116 p53^−/−^) to exclude any p53-dependent effects and performed *ATF2* silencing via RNAi. We discovered a dose-dependent increase in γ-H2AX levels accompanied by apoptosis induction in an ATF2-independent manner (Fig. 6d). Obvious differences in p-ATR^Thr1989^ and p-Chk1^Ser317^ at the protein level among parental, scramble-transfected, and ATF2-siRNA transfected cells were lacking (Fig. 6d). Moreover, 5-FU failed to stimulate the ATF2-p-ATR^Thr1989^ complex in Co-IP (Fig. 6e). Therefore, ATF2 no longer affects cell apoptosis via the ATR/Chk1 pathway when p53 is absent.

**Fig. 7:**
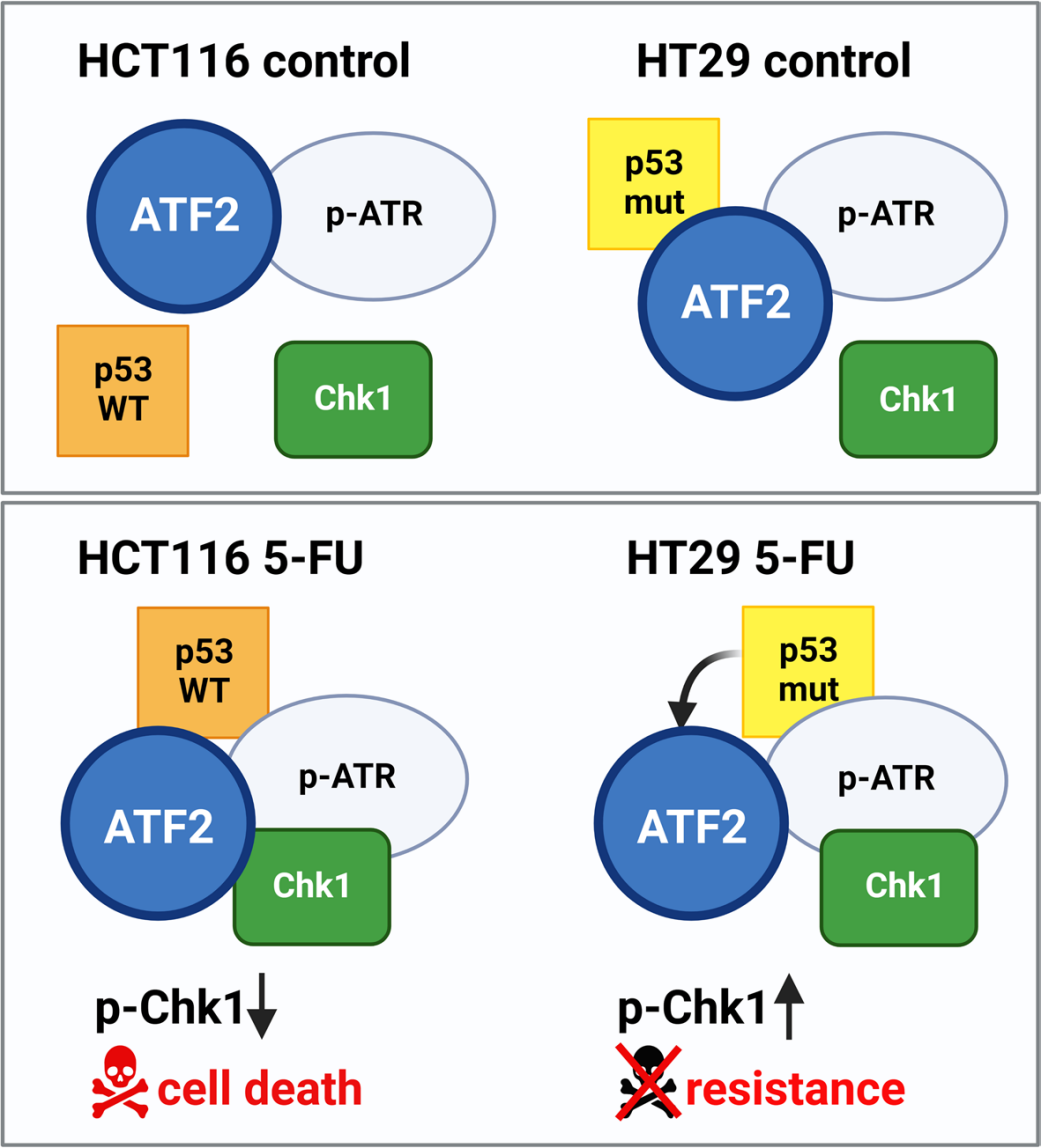
Schematic model of the role of ATF2 in DDR upon 5-FU exposure depending on p53 mutation status. DNA-damaging reagents such as 5-FU induce the DDR pathway. ATR, as a sensor of DNA damage, phosphorylates its downstream target Chk1 upon activation, allowing DNA repair. After 5-FU treatment, ATF2 binds to ATR to suppress the p-ATR/Chk1 protein kinase cascade, triggering apoptosis. Upon ATF2-KO, the interaction of p-ATR/Chk1 is no longer blocked, leading to apoptosis resistance. p53-mutant cells (HT29) show a p53-p-ATR-Chk1-ATF2 complex upon 5-FU exposure. Whereas ATF2 is still in complex with p-ATR, it is released from the complex with mutant p53. Thus, ATF2 cannot inhibitory interact with the p-ATR-Chk1 complex. The resulting changes in complex conformation induce drug resistance accompanied by high p-Chk1 levels

Taken together, we observed different mechanisms upon 5-FU treatment depending on ATF2 expression and p53 mutation status (Fig. 7). In p53-WT/ATF2-WT cells (HCT116), we showed that 5-FU treatment promotes ATF2 binding to ATR, thereby suppressing DNA repair and inducing apoptosis. In p53-WT/ATF2-KO cells, ATR and Chk1 interact, resulting in increased p-Chk1^Ser317^ levels with enhanced DNA damage repair, a mechanism promoting drug resistance.

In p53-mutant cells, we observed a different scenario after 5-FU treatment. In p53-mutant/ATF2-WT cells (HT29), 5-FU treatment led to the binding of both mutant p53 and Chk1 to ATR while displacing ATF2 from the complex. Therefore, ATF2 could not further exert its tumor suppressive effect on the ATR-Chk1 complex, resulting in drug resistance. In p53-mutant/ATF2-KO cells, the complex of mutant p53, ATR, and Chk1 resulted in reduced levels of p-Chk1^Ser317^ and an increase in apoptosis. Thus, the loss of both tumor suppressors, i.e. by ATF2-KO and mutant p53, cannot be compensated by the cells, resulting in cell collapse. Correspondingly, we observed Chk1 addiction in p53-WT/ATF2-KO cells and in p53-mutant/ATF2-WT cells.

## Discussion

Therapy resistance presents a major obstacle in the treatment of colon cancer (CC) patients with 5-fluorouracil (5-FU). Although many promising new regimens, particularly 5-FU-based combination protocols, have been developed, the molecular basis of 5-FU-based resistance is still not completely understood.

The transcription factor ATF2 plays an important role in the response to cellular stress. It is activated by genotoxic compounds and by inflammation, but its role in apoptosis induction is controversial and seems to be dependent on the cell type, the genetic context, and the stimulus [32]. Among the plethora of dysregulated ATF2 target genes, *Jun*, *ATM* and the repair-associated *GADD45* were found. The role of ATF2 in damage repair has already been highlighted in tumor cells treated with ionizing radiation [33]. In an attempt to identify ATF2 target genes that mediate cisplatin-induced damage, the major functional pathway regulated by these genes was the DNA damage response/DNA repair [34, 35].

In a previous paper, we already described in TE7 esophageal cells that oxidative stress-induced apoptosis was enhanced in tumor cells with decreased ATF2 levels. Herein, we have identified the upregulation of p21^WAF1^, an ATF2 target, as the responsible resistance mechanism, suggesting that ATF2 plays an oncogenic role in this scenario [36]. These data are in line with our finding for p53-mutant ATF2-KO cells since TE7 cells do not have a functional p53 [37]. In TE7 cells, we also observed that the downregulation of p-Chk1 at later time points of H_2_O_2_ treatment was accompanied by an increase in caspase 9 and 3 cleavage, verifying induced apoptosis, but the role of the ATR/p-Chk1 cascade has not yet been studied in detail.

Here, we demonstrated a novel role for ATF2 in 5-FU chemotherapy resistance by regulating the DDR pathway in CC, which was independent of ATF2-mediated transcriptional regulation. ATF2 can act as a scaffold protein attenuating ATR-dependent Chk1 phosphorylation and DNA repair. In p53-WT HCT116 cells, an ATF2-negative status was associated with a 5-FU resistance phenotype. This was shown by a reduction in γ-H2AX and cleaved PARP levels, upregulation of the anti-apoptotic protein Bcl-2, and downregulation of the pro-apoptotic protein Bax. Lower levels of caspase 9 cleavage point to resistance against intrinsic apoptosis [38]. As long-term survival in a colony formation assay is associated with therapeutic resistance [39], we could indeed also demonstrate increased 5-FU resistance in HCT116 ATF2-KO cells in such an experimental setting. ATF2-negative cells showed more and larger colonies, suggesting an increased tolerance to such stress conditions. For the CAM model, we chose a pretreatment protocol. Dead cells after 48 h of 5-FU treatment were washed away, and only surviving cells were transplanted using the same cell number for the control and treatment groups. CAM xenografts derived from these surviving and obviously more resistant HCT116 cells showed even reduced ATF2 levels. Obviously, there was a selection for ATF2 low or no expressing cells from the heterogeneous HCT116 cell population upon 5-FU treatment, an interesting finding which cannot be reached by 2D in vitro studies. In accordance with our in vitro data, CAM xenografts derived from HCT116 ATF2-KO cells showed higher p-Chk1^Ser317^ levels. Unexpectedly, there were remarkably higher levels of γ-H2AX in the 3D CAM model, which might represent the higher steady state of active repair or a slowing down in γ-H2AX foci resolution. In this regard, Kato et al. discussed the limited accessibility of dephosphorylation enzymes by an altered chromatin structure [40]. Although this point cannot be clarified, resistant cells might be able to tolerate such high DNA damage.

The DNA damage response (DDR) comprises a panel of cell cycle checkpoints and DNA repair pathways that allow tumor cells to respond to DNA damaging drugs. ATR and Chk1 are the two major kinases that regulate the S-phase checkpoint to cope with replicative stress. The activation of the ATR-Chk1 pathway, as seen by phosphorylation at Ser317 of Chk1 and Thr1989 of ATR, induces cell cycle arrest to give time for repair. Chk1 is a serine/threonine protein kinase activating checkpoint control with cell cycle arrest, DNA repair or apoptosis. Since Chk1 seems to be indispensable for the S-phase checkpoint, Chk1 inhibitors have already successfully entered clinical trials as therapeutic targets. They should potentiate the cytotoxicity of chemotherapeutic drugs such as 5-FU and allow us to provoke failures in DDR in our in vitro approach. Recent data have demonstrated that the prevalence of somatic DDR defects in colorectal cancer ranges between 10 and 30% and predicts worse outcomes and resistance to therapy [41]. Inhibition of Chk1 leads to checkpoint abrogation and diminished repair function, which then drives cells into death. This effect is much stronger when tumor cells are p53-mutant, as we have also shown in our experiments. It is well known that genetic alterations/mutations modify the cellular response to 5-FU-induced damage. Obviously, the ATF2 effects are strongly dependent not only on the experimental stimulus and the tumor type but also on the combination of different mutations in a given cell.

HCT116 and HT29 tumor cells not only differ in p53 status but also exhibit microsatellite instability (MSI) and microsatellite stability (MSS), respectively [42]. It was reported that abrogation of the ATR-Chk1 interaction sensitized CC cells to 5-FU-induced damage but only in mismatch repair (MMR)-deficient cells. A close functional cooperation between the Chk1-controlled checkpoint and the MMR system has been verified in different CC cell lines [43]. Since MMR plays a central role in maintaining the genetic stability of cells during proliferation, a defective MMR system is causally associated with the MSI phenotype. Indeed, we showed that MSI-HCT116 cells commit to apoptosis upon 5-FU treatment in an ATF2-dependent manner. We verified a scaffold function for ATF2 inhibiting the direct action of ATR kinase on its phosphorylation target Chk1. Conversely, ATF2-negative cells are highly resistant in a p53-WT background since the establishment of a stable ATR-Chk1 complex ensures high p-Chk1^Ser317^ levels to effectively repair 5-FU-induced DNA damage. Chk1-inhibited cells fail to initiate correct G2/M checkpoint activation. Interestingly, this accumulation of double strand breaks is not seen in MMR cells, as observed in HT29 cells. Here, Chk1 inhibition did not lead to an overload in DNA damage, while apoptosis was efficiently induced, suggesting the paradox that apoptosis eliminates cells filtered for damage overload as a survival mechanism.

We provided three lines of evidence for the interaction between ATF2 and ATR upon 5-FU exposure: (i) an increased association between ATF2 and p-ATR^Thr1989^ in Co-IP after 5-FU treatment; (ii) positive ATF2-p-ATR^Thr1989^ signals in proximity ligation assay after 5-FU treatment, and (iii) a lower energy level, thus higher binding of the ATF2-ATR complex compared to the Chk1-ATR complex in in silico modelling. Interestingly, a high number of ATF2-p-ATR^Thr1989^ foci were also present in the cytoplasm of the 5-FU-treated tumor cells. This might be connected to the well-described action of both proteins at the mitochondria [44, 45].

Here, we suggest that ATF2 inhibited ATR downstream signaling by directly inhibiting ATR kinase activity. This was supported by the fact that the binding energy markedly increased when ATF2 was docked into the ATR-Chk1 complex, suggesting destabilization of the triple complex. In this complex, ATF2 proved to be close to the ATR kinase domain, and when deleting the active sites of ATF2 and ATR from the molecular structures, the complex became more unstable. Correspondingly, HCT116 ATF2-KO cells showed higher p-Chk1^Ser317^ levels. Of note, there is a difference in the binding of p53-WT to ATR upon 5-FU treatment. Obviously, one tumor suppressor, ATF2, is sufficient to regulate the ATR-Chk1 interaction under physiological conditions. Upon stress, i.e. 5-FU exposure, p53-WT as a second tumor suppressor, supports ATF2 to help in this challenging situation for the cell. We cannot exclude the possibility that p53-WT also triggers the proapoptotic function of cytoplasmic ATR in addition to ATF2’s role in destabilizing the ATR-Chk1 complex. Thus far, it is only speculative if, in addition to the ATF2-mediated attenuated repair by p-Chk1^Ser317^, the apoptosis-inducing function of ATR itself might also contribute to 5-FU-induced cell death.

This mechanism was attenuated when tumor cells had a p53 mutation. In detail, in HT29 p53-mutant cells upon 5-FU treatment, ATF2 and p-ATR^Thr1989^ formed a complex in Co-IP, suggesting that ATF2 might still affect ATR signaling. Mutant p53 formed a novel complex with p-ATR^Thr1989^ upon 5-FU exposure. Mutant p53 and ATF2 already exist in a complex under normal conditions, but ATF2 is released from this complex when cells are treated with 5-FU. In parallel, the formation of an ATR-Chk1 complex was induced but to a lesser extent in ATF2-KO cells. In agreement with this finding, the p-Chk1^Ser317^ levels were lower in ATF2-KO cells. A study by Liu et al. described that mutant p53 blocks ATR kinase activity by inducing the oligomerization of TopBP1 and physical binding with TopBP1 domains to impair ATR activation [46]. We observed that mutant p53 is able to interfere with ATR-mediated Chk1 phosphorylation by stronger binding to p-ATR^Thr1989^ when ATF2 is lost. This might explain the higher sensitivity of HT29 ATF2-KO clones to 5-FU. When both ATF2 and p53 tumor suppressor functions were lost, 5-FU-induced DNA damage overload resulted in cell collapse. Cells that lack functional p53 heavily depend on the G2 checkpoint for DNA repair and survival since the p53-p21 pathway is defective in these cells [47]. Indeed, the inhibition of the G2 checkpoint in p53-deficient cells has been shown to increase sensitivity to DNA damage-inducing agents in vitro [48]. Of note, mutant p53 is not equivalent to p53 loss, since p53-mutant protein might be associated with gain of function and has novel transcriptional targets [49]. In addition, cells lacking functional p53 are strongly dependent on the G2 checkpoint to decide between repair-mediated survival and cell death. In ATF2-negative tumors, two obvious mechanisms, the ATF2-dependent induction of the cell cycle inhibitor p21^WAF1^ and the inhibition of the ATR-Chk1 axis, are abrogated since ATF2 is lost and mutant p53 cannot induce p21^WAF1^ upon stress.

In summary, we describe a novel scaffold-mediated function of ATF2 in regulating 5-FU-induced apoptosis in wild-type p53 tumors. Aggressive CC cells without ATF2 are more resistant to 5-FU due to an increased Chk1-mediated repair function. When Chk1 is diminished, the cells will be sensitized to 5-FU-induced damage. This ATF2-dependent function is abrogated in cells with mutant p53.

## Conclusions

Our findings provide at least two suggestions for daily clinical practice: ATF2 loss induces potentially lethal DNA damage, thereby eliminating p53-mutant cancer treated with 5-FU. Notably, p53 mutations occur at a high frequency in colorectal cancer (CRC) and are found in approximately 70% of stage III CRC patients [50]. Thus, no/low ATF2 expression in tumors could identify patients who are highly sensitive to 5-FU-based treatments when they have a p53 mutation. When combining 5-FU treatment with a Chk1 inhibitor, p53-mutant tumors should go effectively into apoptosis. Otherwise, no/low ATF2 expressing p53-WT tumors would become highly sensitive to 5-FU using checkpoint abrogation with a Chk1 inhibitor. The cells are addicted to Chk1-mediated damage repair, progress through the checkpoint and enter into premature, lethal mitosis.

## Supplementary Information

**Additional file 1: Supplementary Figure S1.** 5-FU treatment activates ATF2 via JNK and does 8 not trigger autophagy induction.** Supplementary Figure S2.** Immunohistochemical evaluations of CAM xenografts stained for ATF2, γ-H2AX, p-Chk1Ser317, and Ki67.** Supplement Figure S3.** 5-FU resistance in ATF2-KO-derived CAM xenografts.** Supplement Figure S4.** Positive and negative controls for Proximity Ligation Assays (PLA).** Supplementary Figure S5.** Complex formation after 5-FU treatment is dependent on the p53 mutation status.** Supplementary Figure S6.** Positive and negative controls for Proximity Ligation Assays (PLA).

## Data Availability

The datasets used and/or analysed during the current study are available from the corresponding author on reasonable request.

## References

[1] Huebner K, Procházka J, Monteiro AC, Mahadevan V, Schneider-Stock R (2019). The activating transcription factor 2: an influencer of cancer progression. Mutagenesis.

[2] Yu T, Li YJ, Bian AH, Zuo HB, Zhu TW, Ji SX (2014). The regulatory role of activating transcription factor 2 in inflammation. Mediators Inflamm.

[3] Li JKH, Lai PF, Tribe RM, Johnson MR (2021). Transcription factors regulated by cAMP in smooth muscle of the myometrium at human parturition. Biochem Soc Trans.

[4] Huebner K, Erlenbach-Wuensch K, Prochazka J, Sheraj I, Hampel C, Mrazkova B (2022). ATF2 loss promotes tumor invasion in colorectal cancer cells via upregulation of cancer driver TROP2. Cell Mol Life Sci.

[5] Ma M, Rodriguez A, Sugimoto K (2020). Activation of ATR-related protein kinase upon DNA damage recognition. Curr Genet.

[6] Allen C, Kurimasa A, Brenneman MA, Chen DJ, Nickoloff JA (2002). DNA-dependent protein kinase suppresses double-strand break-induced and spontaneous homologous recombination. Proc Natl Acad Sci U S A.

[7] Menolfi D, Zha S (2020). ATM, ATR and DNA-PKcs kinases-the lessons from the mouse models: inhibition ≠ deletion. Cell Biosci.

[8] Jackson SP, Bartek J (2009). The DNA-damage response in human biology and disease. Nature.

[9] Pearl LH, Schierz AC, Ward SE, Al-Lazikani B, Pearl FM (2015). Therapeutic opportunities within the DNA damage response. Nat Rev Cancer.

[10] Zhang Y, Hunter T (2014). Roles of Chk1 in cell biology and cancer therapy. Int J Cancer.

[11] Goto H, Natsume T, Kanemaki MT, Kaito A, Wang S, Gabazza EC (2019). Chk1-mediated Cdc25A degradation as a critical mechanism for normal cell cycle progression. J Cell Sci.

[12] King C, Diaz HB, McNeely S, Barnard D, Dempsey J, Blosser W (2015). LY2606368 causes replication catastrophe and antitumor effects through CHK1-dependent mechanisms. Mol Cancer Ther.

[13] Neizer-Ashun F, Bhattacharya R (2021). Reality CHEK: Understanding the biology and clinical potential of CHK1. Cancer Lett.

[14] Marin-Vicente C, Lyutvinskiy Y, Romans Fuertes P, Zubarev RA, Visa N (2013). The effects of 5-fluorouracil on the proteome of colon cancer cells. J Proteome Res.

[15] Kurasaka C, Ogino Y, Sato A (2021). Molecular mechanisms and tumor biological aspects of 5-fluorouracil resistance in HCT116 human colorectal cancer cells. Int J Mol Sci.

[16] Gralewska P, Gajek A, Marczak A, Rogalska A (2020). Participation of the ATR/CHK1 pathway in replicative stress targeted therapy of high-grade ovarian cancer. J Hematol Oncol.

[17] Robinson HM, Jones R, Walker M, Zachos G, Brown R, Cassidy J (2006). Chk1-dependent slowing of S-phase progression protects DT40 B-lymphoma cells against killing by the nucleoside analogue 5-fluorouracil. Oncogene.

[18] Gali-Muhtasib H, Kuester D, Mawrin C, Bajbouj K, Diestel A, Ocker M (2008). Thymoquinone triggers inactivation of the stress response pathway sensor CHEK1 and contributes to apoptosis in colorectal cancer cells. Cancer Res.

[19] Gali-Muhtasib H, Diab-Assaf M, Boltze C, Al-Hmaira J, Hartig R, Roessner A (2004). Thymoquinone extracted from black seed triggers apoptotic cell death in human colorectal cancer cells via a p53-dependent mechanism. Int J Oncol.

[20] Reilly NM, Novara L, Di Nicolantonio F, Bardelli A (2019). Exploiting DNA repair defects in colorectal cancer. Mol Oncol.

[21] Muenzner JK, Kunze P, Lindner P, Polaschek S, Menke K, Eckstein M (2018). Generation and characterization of hepatocellular carcinoma cell lines with enhanced cancer stem cell potential. J Cell Mol Med.

[22] El-Baba C, Mahadevan V, Fahlbusch FB, Mohan SS, Rau TT, Gali-Muhtasib H (2014). Thymoquinone-induced conformational changes of PAK1 interrupt prosurvival MEK-ERK signaling in colorectal cancer. Mol Cancer.

[23] Allalou A, Wählby C (2009). BlobFinder, a tool for fluorescence microscopy image cytometry. Comput Methods Progr Biomed.

[24] Kozakov D, Hall DR, Xia B, Porter KA, Padhorny D, Yueh C (2017). The ClusPro web server for protein-protein docking. Nat Protoc.

[25] Tina KG, Bhadra R, Srinivasan N (2007). PIC: Protein Interactions Calculator. Nucleic Acids Res.

[26] Guex N, Peitsch MC (1997). SWISS-MODEL and the Swiss-PdbViewer: an environment for comparative protein modeling. Electrophoresis.

[27] Tian W, Chen C, Lei X, Zhao J, Liang J (2018). CASTp 30: computed atlas of surface topography of proteins. Nucleic Acids Res.

[28] DeLano WL (2002). Pymol: An open-source molecular graphics tool. CCP4 Newsl Protein Crystallogr.

[29] Ndreshkjana B, Çapci A, Klein V, Chanvorachote P, Muenzner JK, Huebner K (2019). Combination of 5-fluorouracil and thymoquinone targets stem cell gene signature in colorectal cancer cells. Cell Death Dis.

[30] Mani C, Pai S, Papke CM, Palle K, Gmeiner WH (2018). Thymineless death by the fluoropyrimidine polymer F10 involves replication fork collapse and is enhanced by Chk1 inhibition. Neoplasia.

[31] Maiuthed A, Ninsontia C, Erlenbach-Wuensch K, Ndreshkjana B, Muenzner JK, Caliskan A (2018). Cytoplasmic p21 mediates 5-fluorouracil resistance by inhibiting pro-apoptotic Chk2. Cancers (Basel).

[32] Watson G, Ronai ZA, Lau E (2017). ATF2, a paradigm of the multifaceted regulation of transcription factors in biology and disease. Pharmacol Res.

[33] Bhoumik A, Takahashi S, Breitweiser W, Shiloh Y, Jones N, Ronai Z (2005). ATM-dependent phosphorylation of ATF2 is required for the DNA damage response. Mol Cell.

[34] Hayakawa J, Mittal S, Wang Y, Korkmaz KS, Adamson E, English C (2004). Identification of promoters bound by c-Jun/ATF2 during rapid large-scale gene activation following genotoxic stress. Mol Cell.

[35] Hayakawa J, Depatie C, Ohmichi M, Mercola D (2003). The activation of c-Jun NH2-terminal kinase (JNK) by DNA-damaging agents serves to promote drug resistance via activating transcription factor 2 (ATF2)-dependent enhanced DNA repair. J Biol Chem.

[36] Walluscheck D, Poehlmann A, Hartig R, Lendeckel U, Schönfeld P, Hotz-Wagenblatt A (2013). ATF2 knockdown reinforces oxidative stress-induced apoptosis in TE7 cancer cells. J Cell Mol Med.

[37] Barnas C, Martel-Planche G, Furukawa Y, Hollstein M, Montesano R, Hainaut P (1997). Inactivation of the p53 protein in cell lines derived from human esophageal cancers. Int J Cancer.

[38] Bunting KD, Qu CK, Tomasson MH (2012). Molecular-targeted therapies for hematologic malignancies. Adv Hematol.

[39] Bravo-San Pedro JM, Kepp O, Sauvat A, Rello-Varona S, Kroemer G, Senovilla L (2021). Clonogenic assays to detect cell fate in mitotic catastrophe. Methods Mol Biol.

[40] Kato TA, Okayasu R, Bedford JS (2008). Comparison of the induction and disappearance of DNA double strand breaks and gamma-H2AX foci after irradiation of chromosomes in G1-phase or in condensed metaphase cells. Mutat Res.

[41] Tomasini PP, Guecheva TN, Leguisamo NM, Péricart S, Brunac AC, Hoffmann JS (2021). Analyzing the opportunities to target DNA double-strand breaks repair and replicative stress responses to improve therapeutic index of colorectal cancer. Cancers (Basel).

[42] Duldulao MP, Lee W, Le M, Chen Z, Li W, Wang J (2012). Gene expression variations in microsatellite stable and unstable colon cancer cells. J Surg Res.

[43] Jardim MJ, Wang Q, Furumai R, Wakeman T, Goodman BK, Wang XF (2009). Reduced ATR or Chk1 expression leads to chromosome instability and chemosensitization of mismatch repair-deficient colorectal cancer cells. Mol Biol Cell.

[44] Lau E, Ronai ZA (2012). ATF2 - at the crossroad of nuclear and cytosolic functions. J Cell Sci.

[45] Makinwa Y, Cartwright BM, Musich PR, Li Z, Biswas H, Zou Y (2020). PP2A regulates phosphorylation-dependent isomerization of cytoplasmic and mitochondrial-associated ATR by Pin1 in DNA damage responses. Front Cell Dev Biol.

[46] Liu K, Lin FT, Graves JD, Lee YJ, Lin WC (2017). Mutant p53 perturbs DNA replication checkpoint control through TopBP1 and Treslin. Proc Natl Acad Sci U S A.

[47] Leijen S, Beijnen JH, Schellens JH (2010). Abrogation of the G2 checkpoint by inhibition of Wee-1 kinase results in sensitization of p53-deficient tumor cells to DNA-damaging agents. Curr Clin Pharmacol.

[48] Massey AJ (2016). Inhibition of ATR-dependent feedback activation of Chk1 sensitises cancer cells to Chk1 inhibitor monotherapy. Cancer Lett.

[49] Stein Y, Rotter V, Aloni-Grinstein R (2019). Gain-of-function mutant p53: all the roads lead to tumorigenesis. Int J Mol Sci.

[50] Lin PC, Yeh YM, Chan RH, Lin BW, Chen PC, Pan CC (2021). Sequential and co-occurring DNA damage response genetic mutations impact survival in stage III colorectal cancer patients receiving adjuvant oxaliplatin-based chemotherapy. BMC Cancer.

