# Supplementary figures and images for: ATF2 loss promotes 5-FU resistance in colon cancer cells via activation of the ATR-Chk1 damage response pathway
Source: BMC Cancer. 2023 May 27;23:480. doi: 10.1186/s12885-023-10940-0 (PMC10223906; doi:10.1186/s12885-023-10940-0)

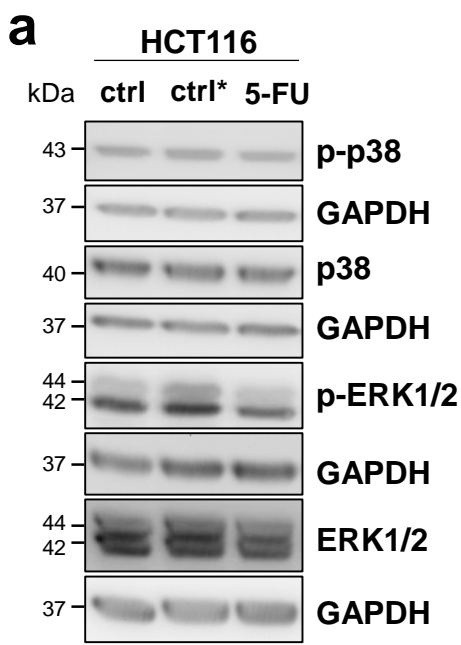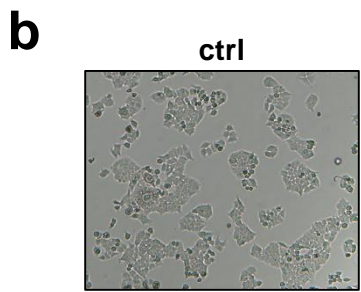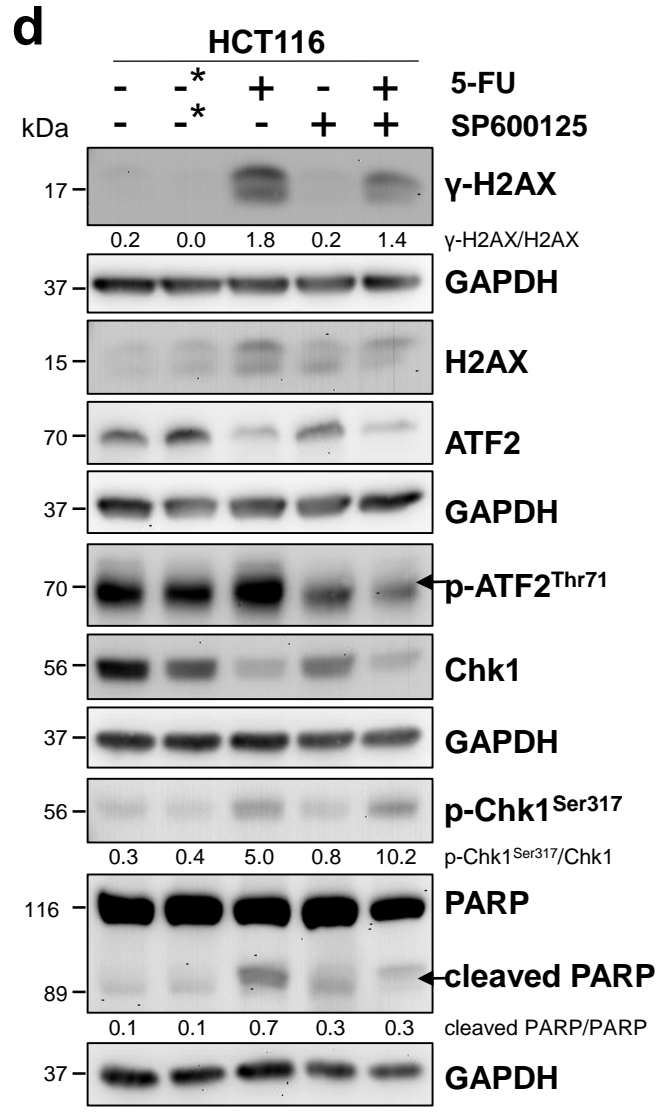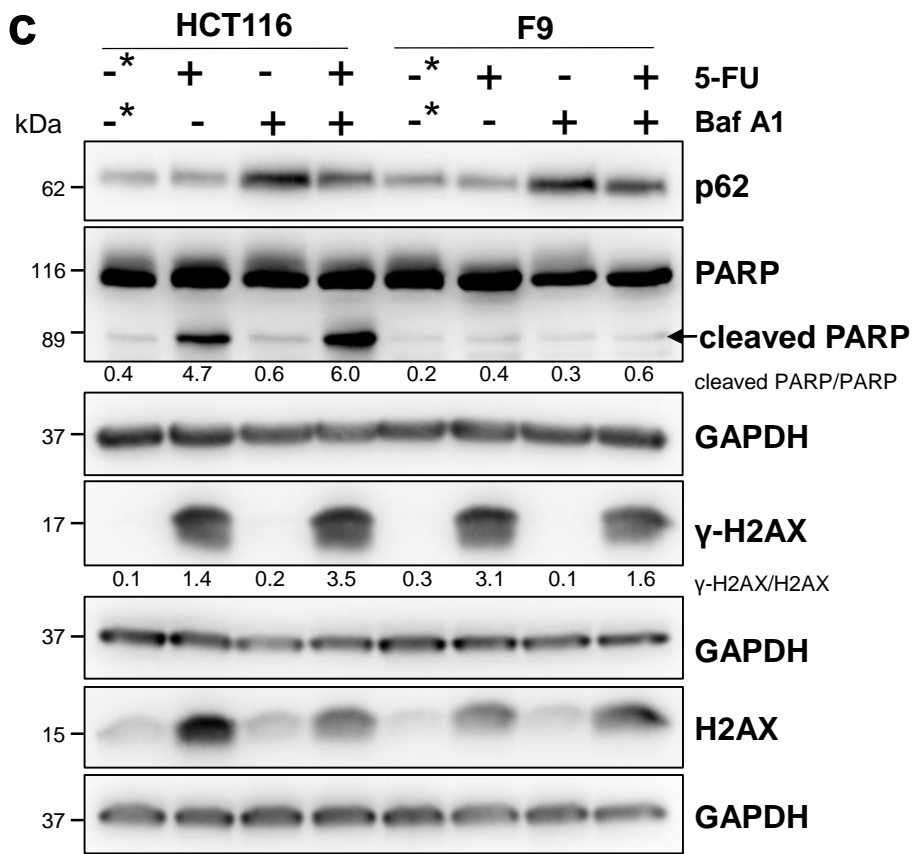

Figure S1

Supplement: Supplementary file 1 — Additional file 1: Supplementary Figure S1. 5-FU treatment activates ATF2 via JNK and does 8 not trigger autophagy induction. Supplementary Figure S2. Immunohistochemical evaluations of CAM xenografts stained for ATF2, γ-H2AX, p-Chk1Ser317, and Ki67. Supplement Figure S3. 5-FU resistance in ATF2-KO-derived CAM xenografts. Supplement Figure S4. Positive and negative controls for Proximity Ligation Assays (PLA). Supplementary Figure S5. Complex formation after 5-FU treatment is dependent on the p53 mutation status. Supplementary Figure S6. Positive and negative controls for Proximity Ligation Assays (PLA). [file 12885_2023_10940_MOESM1_ESM.pdf]
